# Supplementary material for: Overexpression of Renin-B Induces Warburg-like Effects That Are Associated with Increased AKT/mTOR Signaling
Source: Cells. 2022 Apr 26;11(9):1459. doi: 10.3390/cells11091459 (PMC9103744; doi:10.3390/cells11091459)
Supplement: Supplementary file 1 [file cells-11-01459-s001.zip › cells-1619160 supplementary Figure S1.zip!/cells-1619160 supplementary Figure S1.pdf]

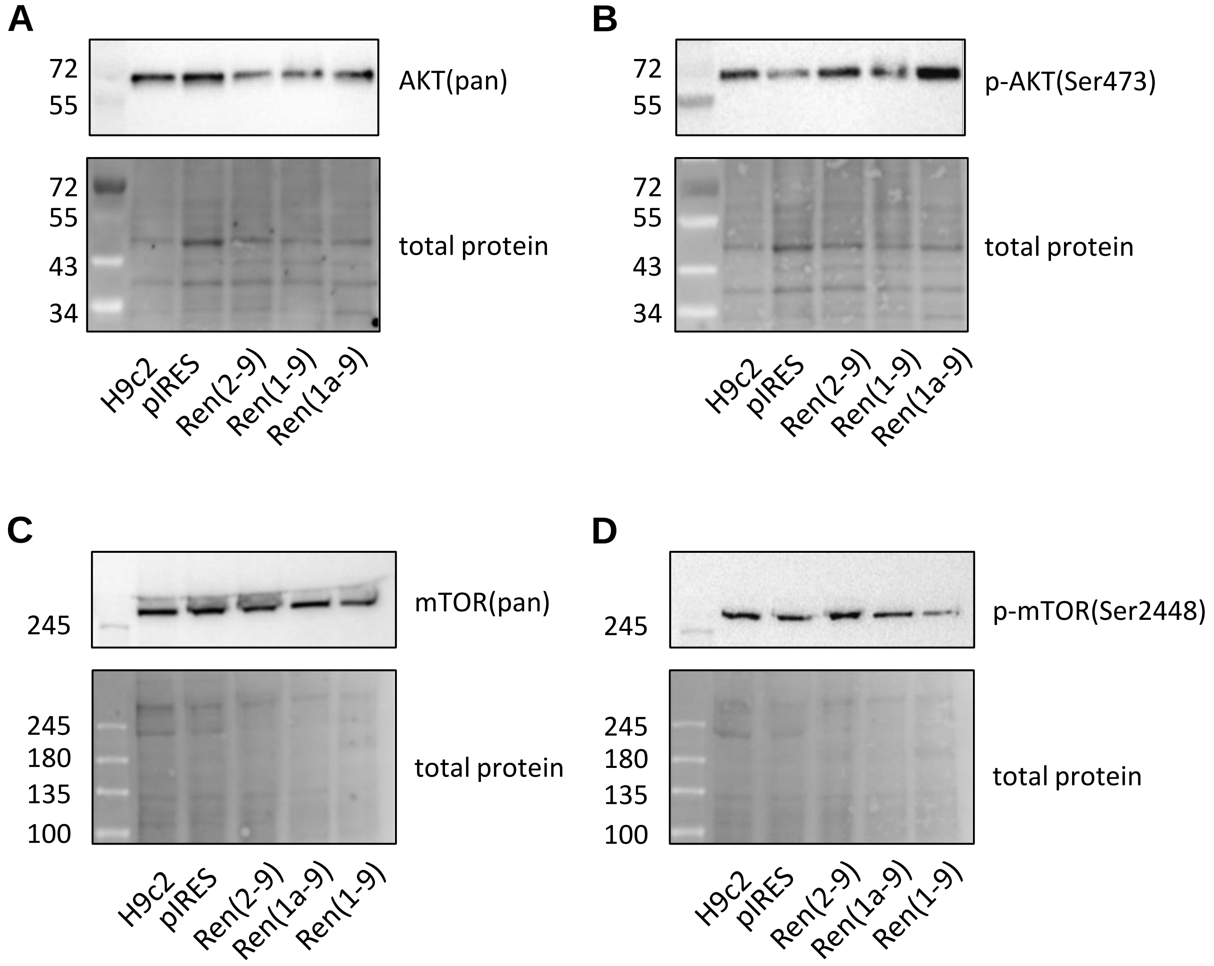

Figure S1: Representative western blot images of the protein abundances of the kinases AKT and mTOR complementing figure 7. Protein levels of total and phosphorylated AKT (**A** and **B** at top) and mTOR (**C** and **D** at top) as well as total protein (**A-D** at bottom) were detected by western blot analysis of pIRES control as well as Ren(1a-9) and Ren(2-9) overexpressing H9c2 cells. In addition, protein levels of the kinases AKT and mTOR from untransfected H9c2 cells and Ren(1-9) overexpressing H9c2 cells were also examined. Since these groups were not analyzed in the transcriptome study, they were also not considered for statistical analysis in the western blot study.
